# Analysis of immunogenic cell death in periodontitis based on scRNA-seq and bulk RNA-seq data

**DOI:** 10.3389/fimmu.2024.1438998

**Published:** 2024-11-01

**Authors:** Erli Wu, Xuan Yin, Feng Liang, Xianqing Zhou, Jiamin Hu, Wanting Yuan, Feihan Gu, Jingxin Zhao, Ziyang Gao, Ming Cheng, Shouxiang Yang, Lei Zhang, Qingqing Wang, Xiaoyu Sun, Wei Shao

**Affiliations:** ^1^ Key Laboratory. of Oral Diseases Research of Anhui Province, College & Hospital of Stomatology, Anhui Medical University, Hefei, China; ^2^ Department of Periodontology, Anhui Stomatology Hospital Affiliated to Anhui Medical University, Hefei, China; ^3^ Department of Microbiology and Parasitology, Anhui Provincial Laboratory of Pathogen Biology, School of Basic Medical Sciences, Anhui Medical University, Hefei, Anhui, China

**Keywords:** immunogenic cell death (ICD), periodontitis, fibroblasts, machine learning, biomarker

## Abstract

**Background:**

Recent studies have suggested that cell death may be involved in bone loss or the resolution of inflammation in periodontitis. Immunogenic cell death (ICD), a recently identified cell death pathway, may be involved in the development of this disease.

**Methods:**

By analyzing single-cell RNA sequencing (scRNA-seq) for periodontitis and scoring gene set activity, we identified cell populations associated with ICD, which were further verified by qPCR, enzyme linked immunosorbent assay (ELISA) and immunofluorescence (IF) staining. By combining the bulk transcriptome and applying machine learning methods, we identified several potential ICD-related hub genes, which were then used to build diagnostic models. Subsequently, consensus clustering analysis was performed to identify ICD-associated subtypes, and multiple bioinformatics algorithms were used to investigate differences in immune cells and pathways between subtypes. Finally, qPCR and immunohistochemical staining were performed to validate the accuracy of the models.

**Results:**

Single-cell gene set activity analysis found that in non-immune cells, fibroblasts had a higher ICD activity score, and KEGG results showed that fibroblasts were enriched in a variety of ICD-related pathways. qPCR, Elisa and IF further verified the accuracy of the results. From the bulk transcriptome, we identified 11 differentially expressed genes (DEGs) associated with ICD, and machine learning methods further identified 5 hub genes associated with ICD. Consensus cluster analysis based on these 5 genes showed that there were differences in immune cells and immune functions among subtypes associated with ICD. Finally, qPCR and immunohistochemistry confirmed the ability of these five genes as biomarkers for the diagnosis of periodontitis.

**Conclusion:**

Fibroblasts may be the main cell source of ICD in periodontitis. Adaptive immune responses driven by ICD may be one of the pathogenesis of periodontitis. Five key genes associated with ICD (ENTPD1, TLR4, LY96, PRF1 and P2RX7) may be diagnostic biomarkers of periodontitis and future therapeutic targets.

## 1 Introduction

Periodontitis is an inflammatory disease of the gingiva characterized by periodontal tissue destruction. The disease is mainly manifested by the deterioration of the alveolar bone, cementum, gums, and periodontal ligaments, which eventually leads to tooth loss (1). In addition, periodontitis can also lead to an increased risk of a number of systemic diseases, such as diabetes, rheumatoid arthritis (RA), cancer, neurological disorders and cardiovascular problems (2–6). These symptoms place a huge burden on patients and place considerable stress on the families and society of affected patients. Periodontitis has consequently emerged as a major worldwide public health issue. According to reports, between 20% and 50% of people worldwide have periodontal disease. It is among the primary reasons why teeth fall out in adults. Furthermore, between 1990 and 2010, the worldwide prevalence of periodontal disease rose by 57.3% (7). Simultaneously, the growing aging population is predicted to contribute to the global prevalence of periodontal disease in the upcoming years (8). Therefore, exploring the pathogenesis of periodontitis is of great significance for the treatment of periodontitis.

The causes of periodontitis are multifactorial, including bacterial infection and its triggering of host immune response, oxidative stress, and cell death, among others (9, 10). Research on cell death has been a hot topic in recent years. One of the early findings was the loss of fibroblasts in humans during the transition from gingivitis to periodontitis (11). DNA microarray analysis showed that the expression of apoptosis-related genes in gingival tissue and peripheral blood mononuclear cells in patients with chronic periodontitis was up-regulated compared with that in the healthy group (12, 13). Animal studies have shown that *Porphyromonas gingivalis* (*P. gingivalis*)-mediated fibroblast apoptosis was significantly reduced in TNF receptor deficient mice, suggesting that bone loss and soft tissue destruction associated with *P. gingivalis* infection may be due to host-derived factors rather than direct effects of bacterial products (14). Developmental endothelial locus-1 (DEL-1), a secreted multifunctional protein, can promote the resolution of inflammation in periodontitis by promoting macrophage phagocytosis of apoptotic neutrophils (15). In addition, *Fusobacterium nucleatum* (*F. nucleatum*) can induce apical periodontitis by triggering pro-inflammatory cell death through Z-DNA binding protein 1 (ZBP1) (16). Furthermore, Lu et al. (17) found that ferroptosis triggered by the pathogen lipopolysaccharide (LPS) mainly occurred in fibroblasts, and inhibiting ferroptosis in fibroblasts alleviated tissue damage and bone loss induced by periodontitis by inhibiting IL-6. Zhang et al. (18) found that necrotic apoptosis of gingival fibroblasts triggered by LPS aggravated gingival inflammation and alveolar bone loss. Necrotic apoptosis inhibitors attenuate this process by regulating THP-1 macrophage migration and polarization. These studies suggest that cell death caused by oral bacteria can drive host immune responses that mediate inflammation and bone loss in periodontitis.

Immunogenic cell death (ICD) is a type of regulatory cell death that leads to the release of damage-associated molecular patterns (DAMPs), including heat shock protein (HSP), adenosine triphosphate (ATP), calreticulin, and high mobility basal box 1 (HMGB1) proteins, which are subsequently recognized by pattern-recognition receptors (PRRs) on the surface of antigen-presenting cells (APC) to activate innate and adaptive immune responses (19, 20). Studies have shown that ICD is crucial to the pathophysiology of numerous non-oncological illnesses, including COVID-19 (21), atherosclerosis, Alzheimer’s disease (22), and severe acute pancreatitis (23). Additionally, some evidence also suggests that ICD may be involved in the development of periodontitis. For instance, some periodontal pathogens, such as *P. gingivalis* or *F. nucleatum*, are associated with DAMP release, which can trigger immune responses and antigenic properties by activating host cells expressing PRRs (24). Moreover, ICD-induced DAMPs such as HMGB1 are increased in periodontal inflammatory tissue and gingival crevicular fluid (GCF), and HMGB1 can stimulate the secretion of pro-inflammatory mediators and cytokines, thereby initiating an adaptive immune response (25, 26). Anti-HMGB1 neutralizing antibodies can reduce periodontal inflammation and bone resorption in mouse periodontitis models (26). Other DAMPs such as HSP70, cyclophilin A, amyloid beta, high mobility group nucleosomal-binding domain 2 (HMGN2) and IL-1 were also found to be highly expressed at the site of periodontitis (27). Also, extracellular ATP is a key regulator of alveolar bone loss in periodontitis, and by controlling the interaction of extracellular ATP with its cytosolic purinergic receptors, such as the P2X7 receptor (P2X7R), bone loss in periodontitis will be significantly reduced (28). Based on the above studies, we speculated that ICD may be involved in bone loss in periodontitis. Nevertheless, more investigation is needed to confirm this.

This study aimed to provide a description of the function of ICD in the course of periodontitis from two perspectives. First of all, in terms of the single-cell level, we identified the main cells in which ICD occurs and took multiple approaches to validate them. Secondly, at the bulk RNA-seq level, we screened ICD-related DEGs and constructed an ICD gene-related model for the diagnosis of periodontitis through two machine learning methods. Afterwards, based on model gene expressions, samples with periodontitis were divided into two separate types. These subtypes’ immune mechanisms and differences were identified through a thorough analysis of the immune infiltration subtypes, which contributed significantly to our understanding of the pathophysiology of periodontitis. Finally, we validated the genes involved in the ICD model using qPCR and immunohistochemistry.

## 2 Materials and methods

### 2.1 Data acquisition

scRNA-seq dataset (GSE164241) and bulk RNA-seq datasets (GSE16134 and GSE10334) were retrieved for analysis from the GEO database (https://www.ncbi.nlm.nih.gov/geo/). GSE164241 was used for scRNA analysis as a single-cell dataset consisting of 13 samples of healthy and 8 samples of inflamed gingiva. For the RNA-seq datasets, 310 samples were included in the GSE16134 dataset (241 periodontitis cases and 69 healthy controls), while 247 samples were included in the GSE10334 dataset (183 periodontitis cases and 64 healthy controls). We used the GSE16134 dataset as the training dataset and the GSE10334 dataset as the validation dataset. Moreover, 34 ICD-related genes from previous studies were also included in this study (29).

### 2.2 scRNA sequencing data processing

To read scRNA-seq data (GSE164241), the Seurat software (version 4.3.1) was utilized for preprocessing. To ensure that the majority of cells were included in the dataset, we performed data quality control and excluded cells with gene expression levels below 200 genes or more than 4,000 genes, as well as cells with mitochondrial gene expression levels above 20%. Next, we apply the LogNormalize method to normalize the data. The “FindVariableFeatures” program was utilized to find the top 2000 genes that exhibit high variability. The data was adjusted using the “harmony” package to eliminate batch effects between samples (30). For the purposes of downscaling and clustering identification, we used Unified Flow Approximation and Projection (UMAP) and the top 20 principal components (31). To identify marker genes in cell subsets, the highly expressed genes within each subgroup were determined using the “FindAllMarkers” function, with logFC > 0.25 and min.pct > 0.25 as thresholds (32). Subsequently, the clusters identified were annotated according to the previous literature and known cell surface markers. In order to find the cell subpopulations most associated with ICD, we used four algorithms, including AUCell, UCell, ssGSEA, and AddModuleScore, to score the activity of gene sets. In addition, the use of the “ggplot2” R package to visually represent each cell’s score helps to identify clusters characterized by active gene set profiles.

### 2.3 Cell-cell communication analysis

We studied the signals and pathways related to ICD by estimating intercellular communication networks from scRNA-seq data using the R “CellChat” program (30). CellChat uses a ligand-receptor interaction database to detect intercellular communication at the pathway level and calculate clustered cell communication networks.

### 2.4 Identification of DEGs

We compared the expression of 34 ICD-related genes between periodontitis cases and control samples using the “limma” program in R. LogFC > 0.3 and adjusted p values <0.05 were used as screening criteria for DEGs. Heatmaps and boxplots were performed to visualize different results.

### 2.5 Gene ontology and Kyoto encyclopedia of genes and genomes analyses

We used the R package “clusterprofiler” to perform pathway enrichment analysis using the Kyoto Encyclopedia of Genes and Genomes (KEGG) and gene ontology (GO) to examine the functional abundance of the DEGs of bulk RNA-seq data. Also, KEGG analysis was performed on DEGs (logFC > 0.25, p < 0.05) between fibroblast clusters and other non-immune cell clusters in GSE164241. Results with p <0.05 were deemed significant (33).

### 2.6 Immunoinfiltration analysis

We evaluated immune infiltration patterns and immune function in periodontitis and normal samples using the ssGSEA method. The percentage of different types of immune cell infiltration and immune function was displayed using a heatmap and boxplot. A statistical significance level of p<0.05 was determined using the Wilcoxon rank sum test.

### 2.7 Establishment of a prediction model

To further screen for pivotal genes associated with ICD in periodontitis, we used least absolute shrinkage and selection operator (LASSO) regression and the random forest (RF) algorithm to identify potential feature genes and use them to build models. The “randomForest” package in R was utilized to ascertain the significance of each gene and identify the top 5 genes for RF analysis. LASSO regression analysis was carried out using the R “glmnet” package. Using the R “mlr3verse” package (https://CRAN.R-project.org/package=mlr3verse), we implemented seven machine learning methods to create a model in order to evaluate the possible signature genes’ diagnostic potential. Receiver operating characteristic (ROC) curve analysis in the training and validation sets yielded area under the curve (AUC) values, which were used to assess the predictive performance of the seven models.

### 2.8 Candidate biomarker expression levels and diagnostic value

The “limma” and “ggpubr” R packages were used in the study to compare and analyze the expression levels of potential biomarkers in the training and validation sets, respectively. Using the “pROC” software, ROC curve analysis was carried out simultaneously for each core gene, and the AUC of the 95% confidence interval (CI) was computed (34). The AUC value, which is near 1, indicates that a gene’s diagnostic accuracy is high and hence serves as a proxy for its diagnostic value.

### 2.9 Gene set enrichment analysis and interaction network of model genes

GSEA is a tool used to elucidate the biological significance of functional gene definitions. Genes that differed in expression between the two clusters were extracted for examination after the data were divided into two groups according to the model’s median gene expression value. To create a consistent enrichment score for every analysis, the genomes were ordered 1,000 times. FDR < 0.05 was used to determine significant enrichment. We assessed changes in the KEGG pathway in each gene using GSEA. In order to build an interaction network associated with ICD and assess the function of these ICD-related genes, GENEMANIA (http://genemania.org/search/), GO, and KEGG were utilized.

### 2.10 Identification of ICD-related subtypes

Using the “ConsensusClusterPlus” package in R, we carried out an unsupervised cluster analysis, dividing periodontitis patient samples in GSE16134 into different groups according to 5 predicted ICD-related genes using a 1,000-loop k-means technique. We integrated a consensus matrix, a cumulative distribution function (PDF) curve, and a consistent cluster score to obtain an ideal number of clusters. The sample distribution of each cluster was then assessed using principal component analysis (PCA). Boxplots and heatmaps were used to show the expression of ICD-related genes in subgroups. Furthermore, we computed the differences in immune cell infiltration and immune functions among ICD-related subtypes to investigate the immune involvement of ICD in the development of periodontitis. GSVA and GSEA were used to explore potential biological pathways and processes associated with “c2.cp.kegg.v7.4.symbols.gmt” between different subtypes.

### 2.11 Cell culture and treatments

The cervical gingiva of the clinically extracted third molar was excised for the extraction of primary gingival fibroblasts. For the cultivation of cells, Dulbecco Modified Eagle Medium (DMEM) supplemented with 10% fetal bovine serum, 100 U/mL penicillin/streptomycin at 37°C, and 5% carbon dioxide was utilized. After 7 days, primary cells began to migrate outward from the periodontal ligament tissue. They are passed through and expanded to ensure there are enough cells for the experiment. Cells from generations 4 to 8 were used. For subsequent tests, the cells were treated with *P. gingivalis* LPS for six hours after the cell density reached 80%.

### 2.12 Enzyme linked immunosorbent assay

In accordance with the manufacturer’s guidelines, a commercial kit of HMGB1 (human ELISA kit SEKH-0409-48T) was used to analyze the cell supernatant samples by ELISA.

### 2.13 ATP quantification

Following the manufacturer’s instructions, the ATP content assay kit (BC0300) was used to detect changes in the extracellular ATP content.

### 2.14 Collection of tissue samples

Five healthy gingiva and five inflammatory gingiva were collected from healthy volunteers and periodontitis patients, respectively, from the Affiliated Stomatology Hospital of Anhui Medical University (Hefei, Anhui Province). The study was examined and approved by the Ethics Committee of the Affiliated Stomatology Hospital of Anhui Medical University (Ethics number: 2021006). Written informed consent was obtained from each subject.

### 2.15 RNA extraction and quantitative real-time PCR

Tissue total RNA was extracted using the TRIzol reagent (Thermo Fisher Scientific, USA). Using Takara’s Prime Script RT premix, total RNA extracted from tissue was reverse-transcribed into cDNA. qRT-PCR was carried out in compliance with the manufacturer’s experimental procedure using the CFX96 Touch real-time fluorescence quantitative PCR detection system (Bio-Rad, Hercules, CA, United States). The 2^△△^CT method was used to calculate the relative expression level, with GAPDH acting as the internal reference. Every experiment was performed more than three times. The qRT-PCR primer sequences were given in **Supplementary Table 1**.

### 2.16 Immunofluorescence assay

The sample of gingival tissue was divided into 4μm pieces and fixed in paraffin wax. The sections were dewaxed, the antigen was removed, and then the primary antibody (HMGB1) was incubated at 4°C for an overnight period. After staining the sections for one hour at room temperature using a secondary antibody that identified the main antibody, we stained them for three minutes with DAPI. An imaging Zeiss 800 laser scanning confocal microscope was used to capture images of the stained slices.

### 2.17 Immunohistochemistry

We fixed the collected gum tissues in formalin, embedded them in paraffin wax and extracted the antigens after they were sliced and removed. Goat serum was applied to the slide and incubated with the antibody. Finally, we stained the tissue with DAB (Servicebio) and then restained it with hematoxylin. Then, the image processing software (ImageJ v 1.48) is used for image acquisition and processing.

### 2.18 Statistical analysis

The t-unpaired test was selected for the qPCR statistical testing method and the data were presented as means +/- standard deviation. R version 4.3.1 was used for all of the statistical analyses that were addressed before. A significance level of p <0.05 was applied.

## 3 Results

### 3.1 scRNA-seq data processing

**Figure 1:**
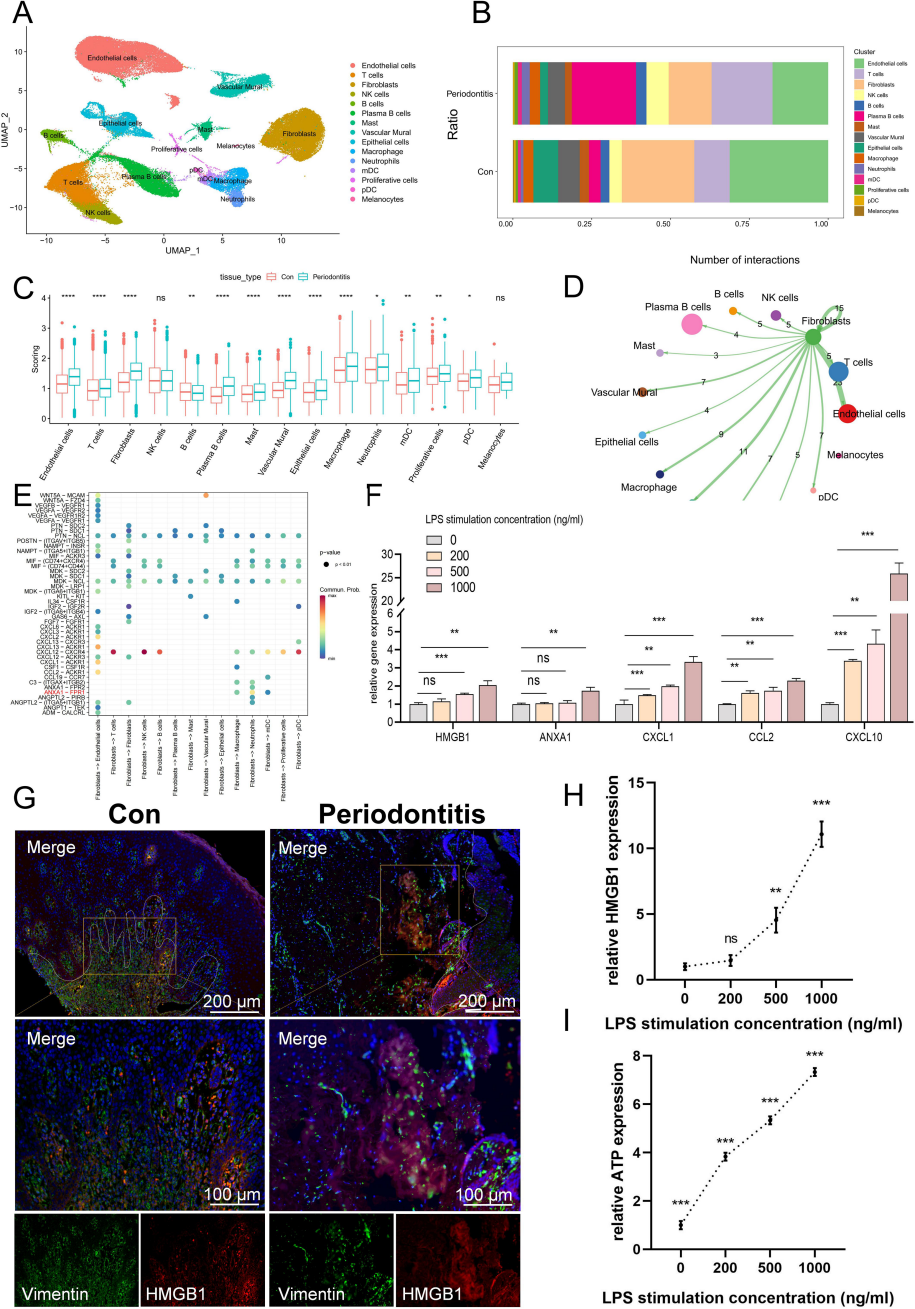
scRNA-seq data processing and identification of the cell types associated with ICD. **(A)** A UMAP of 15 cell clusters. **(B)** Different cell clusters distribution ratio between con group and periodontitis disease group. **(C)** Cell subgroup ICD-related gene set scoring. **(D)** Cell communication showing the results of fibroblast interactions with other cells. **(E)** Bubble plot showing important ligand-receptor pairs between fibroblasts and other cells. **(F)** The mRNA expression levels of DAMP-related molecules associated with ICD development in fibroblasts. **(G)** Immunofluorescence staining of HMGB1, in which vimentin positive represents fibroblasts. **(H)** ELISA results of HMGB1 production at different concentrations after LPS stimulation. **(I)** ATP content in extracellular supernatant at different concentrations after LPS stimulation. ICD, Immunogenic cell death; UMAP, Uniform Manifold Approximation and Projection; DAMP, damage-associated molecular pattern; ELISA, enzyme-linked immunosorbent assay; ATP, adenosine triphosphate; Con, Control.

There were 92556 cells in the scRNA sequencing dataset after the data was filtered, comprising 56,054 cells from control subjects and 36,502 cells from periodontitis patients (**Supplementary Figure 1A**, **Supplementary Table 2**). The annotation of cell surface markers yielded a total of 15 distinct cell types, including endothelial cells, NK cells, T cells, B cells, fibroblasts, plasma B cells, vascular murals, epithelial cells, neutrophils, macrophages, myeloid dendritic cells (mDCs), plasmacytoid dendritic cells (pDCs), mast cells, proliferative cells and melanocytes (**Figure 1A**). The expression of recognized lineage markers in 15 main cell clusters was displayed in **Supplementary Figure 1B**. **Figure 1B** displayed the relative amounts of various cell types in periodontitis samples and normal samples, and it was found that the proportion of fibroblasts decreased significantly in periodontitis samples, suggesting that it may be a key factor in the etiology of the disease.

### 3.2 Identification of the major cells in which ICD occurs

Subsequently, we calculated the activity of the ICD-associated gene set using four methods and found that fibroblasts scored relatively high among non-immune cells and differed between the normal and disease groups (**Figure 1C**). Also, KEGG was used to compare the pathways of fibroblasts and other non-immune cells, and it was found that fibroblasts enriched a variety of ICD-related pathways, such as toll-like receptor signaling pathway, NF-κB, TNF, NOD-like receptors (**Supplementary Tables 3**-**6**, **Supplementary Figure 2A–D**). When the host is exposed to periodontal disease-causing bacteria, the toll-like receptor, a PRR, detects pathogens and triggers the host’s innate immune response and adaptive immunity (35). Similarly, NOD-like receptors can recognize DAMP, which can induce activation of downstream signaling pathways via signals emitted by PRRs, thereby modulating general pro-inflammatory responses (24). The results of cell communication indicated that fibroblasts can activate a variety of APCs, such as mDC and macrophages, to activate the immune response under inflammatory conditions (**Figure 1D**). Moreover, fibroblasts can activate the mDC surface receptor Formyl peptide receptor-1 (FPR1) by releasing Annexin A1 (ANXA1), which was a DAMP molecule closely related to the ICD (**Figure 1E**) (36). Next, we selected several DAMPs-related molecules that were closely related to ICD occurrence for qPCR validation. The results showed that HMGB1 and ANXA1 expression increased in LPS-treated fibroblasts (**Figure 1F**). Meanwhile, the expression of CXCL1, CCL2 and CXCL10 was also increased in LPS-treated fibroblasts, and they can promote the occurrence of ICD by recruiting T cells (36). HMGB1 was further used for IF staining, for it is one of the important DAMPs associated with ICD, and the results showed that in normal fibroblasts, HMGB1 was located in the nucleus, whereas it was released extracellularly in LPS-treated fibroblasts (**Figure 1G**). ELISA results further showed that the content of HMGB1 in extracellular supernatant increased with the increase of LPS stimulation concentration (**Figure 1H**). In addition, the ATP content in the extracellular supernatant of LPS-treated fibroblasts was also increased compared to the normal group (**Figure 1I**). These findings indicated that fibroblasts may be implicated in the occurrence and progression of ICD.

### 3.3 Identification of ICD-related DEGs

**Figure 2:**
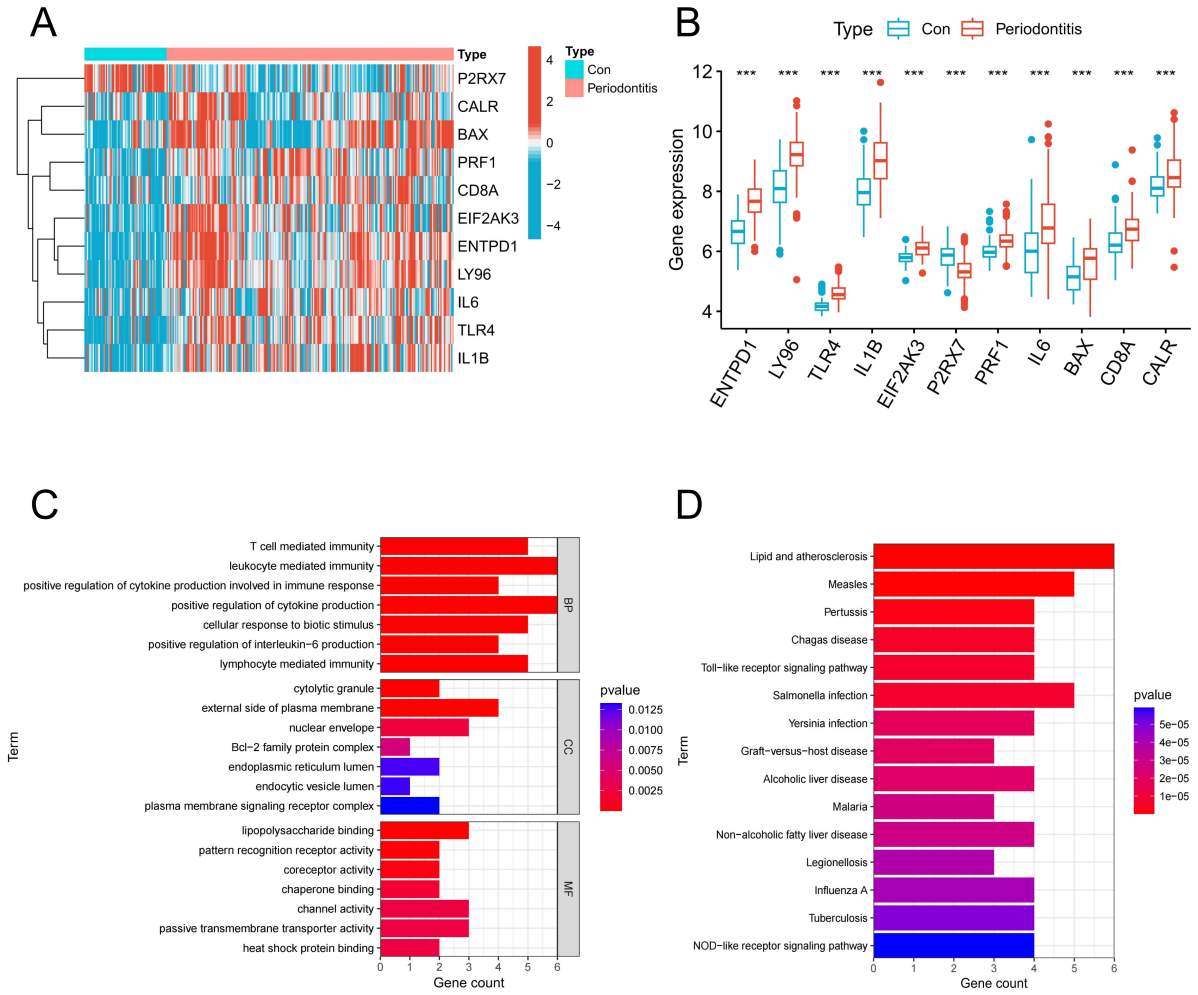
Identification of ICD-associated DEGs. **(A)** Heatmap of ICD-related DEGs in the control group and periodontitis group. **(B)** Expression of ICD-related DEGs in the control group and periodontitis group. **(C)** GO enrichment of ICD-related DEGs. **(D)** KEGG enrichment of ICD-related DEGs. DEGs, differentially expressed genes; ICD, Immunogenic cell death.

Analysis of bulk transcriptome data revealed that 11 genes linked to ICD were expressed differently in the periodontitis group compared to the control group. **Figures 2A, B** illustrated the expression of these 11 genes in periodontitis, with 10 genes up-regulated and 1 genes down-regulated. According to GO findings, BP was primarily linked to leukocyte mediated immunity, T mediated immunity, and the production of IL-6, whereas CC was mostly linked to the cytolytic granule, the external side of the plasma membrane and the Bcl-2 family protein complex; MF class has high levels of passive transmembrane transporter activity, heat shock protein binding and pattern recognition receptor activity (**Figure 2C**). Based on KEGG, these genes were associated with the ICD-related NOD-like receptor signaling pathway and toll-like receptor signaling pathway (**Figure 2D**).

### 3.4 Immune infiltration analysis

**Figure 3:**
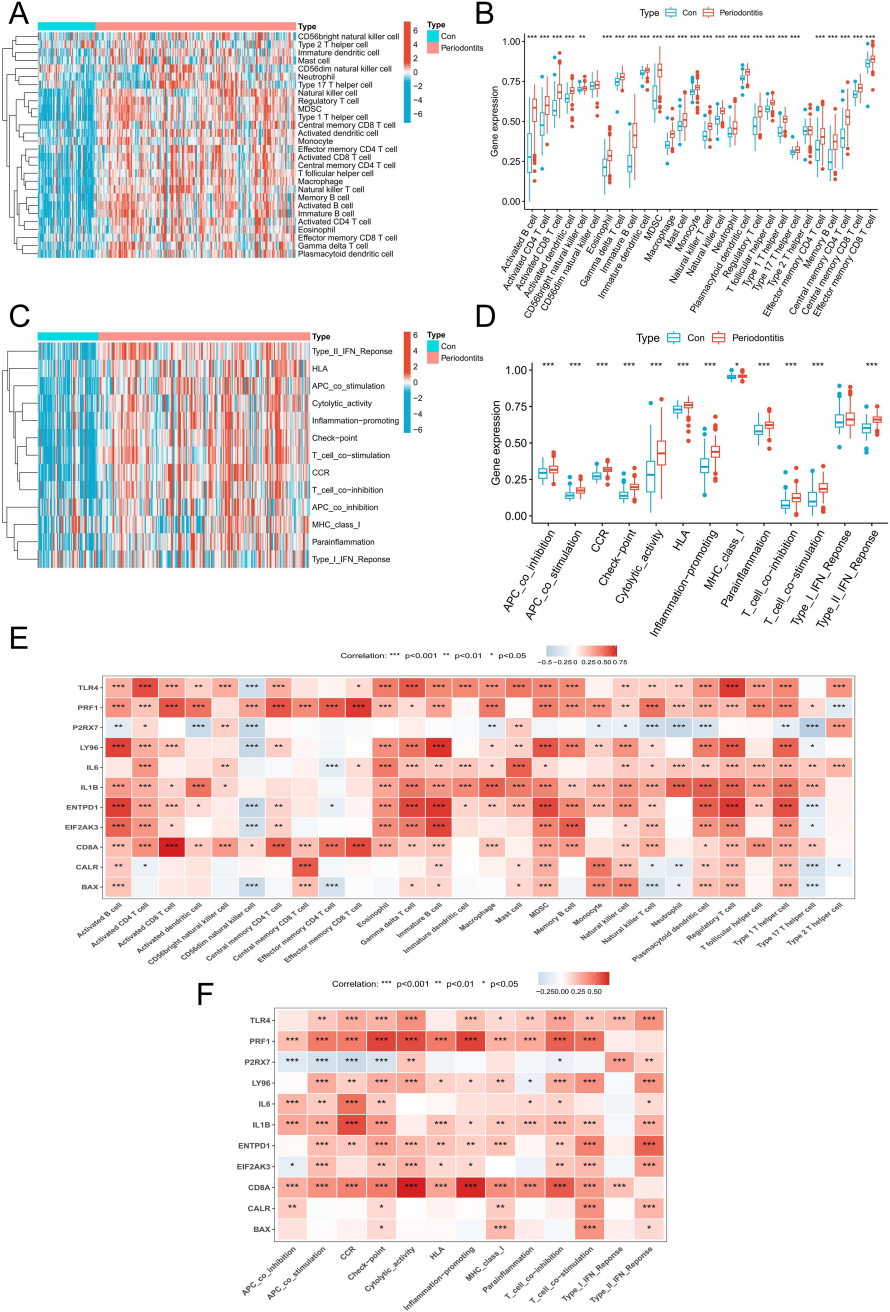
Immune infiltration analysis. **(A)** A heatmap of the distribution of 28 immune cells in normal samples and periodontitis samples. **(B)** A boxplot of the distribution of 28 immune cells in normal samples and periodontitis samples. **(C)** A heatmap of the distribution of immune functions in normal samples and periodontitis samples. **(D)** A boxplot of the distribution of immune functions in normal samples and periodontitis samples. **(E)** The relationship between DEGs associated with ICD and immune cell infiltration in the periodontitis. **(F)** The relationship between DEGs associated with ICD and immune functions in the periodontitis. DEGs, differentially expressed genes; ICD, Immunogenic cell death.

An algorithm based on ssGSEA was used in order to confirm the role of immune responses in periodontitis. In comparison to the control group, the periodontitis group exhibited a considerable increase in the percentage of different immune cells, particularly T cells and diverse APCs (**Figures 3A, B**). Also, the periodontitis group showed upregulation of multiple immune functions, including APC co-stimulation, CCR, immune checkpoint and MHC class I (**Figures 3C, D**). Afterwards, we investigated the association of these 11 DEGs with immune cells and functions and found that they were significantly associated with multiple immune cells and functions (**Figures 3E, F**). These findings imply that ICD-related DEGs were intimately connected to the immune response.

### 3.5 Disease prediction model results

**Figure 4:**
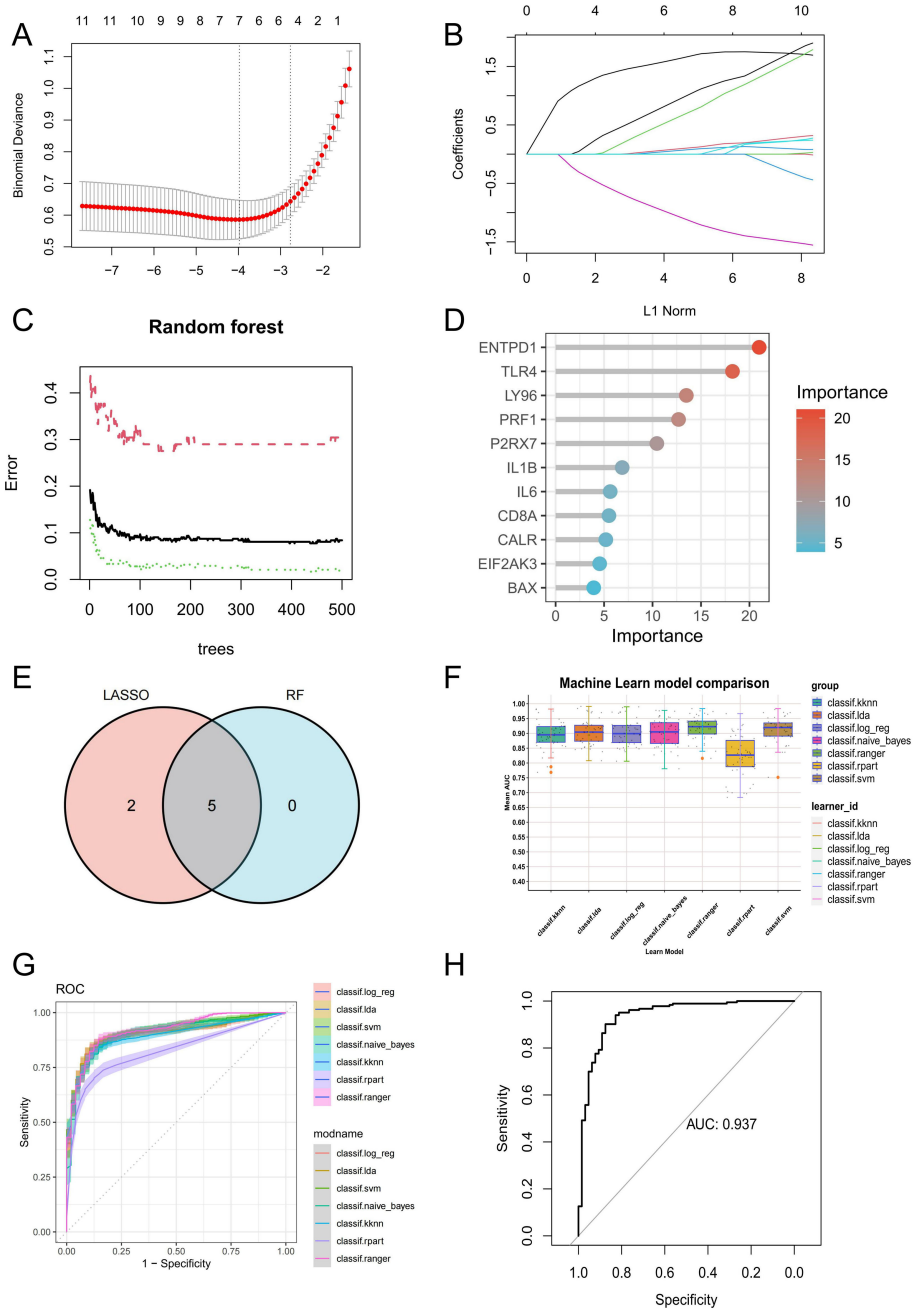
Identification of potential ICD-related diagnostic genes and construction of models. **(A, B)** Establishment of the LASSO model. **(C, D)** The relative importance of potential feature genes was calculated in random forest (RF) algorithm (Top 5 genes’ importance > 2). **(E)** Venn diagram showing the overlap between the two algorithms. **(F)** Seven machine learning algorithms were utilized for model construction. **(G)** The ROC values of all seven algorithms in the training group. **(H)** The ROC scores of the ranger model were presented in the test group. ICD, Immunogenic cell death.

**Figure 5:**
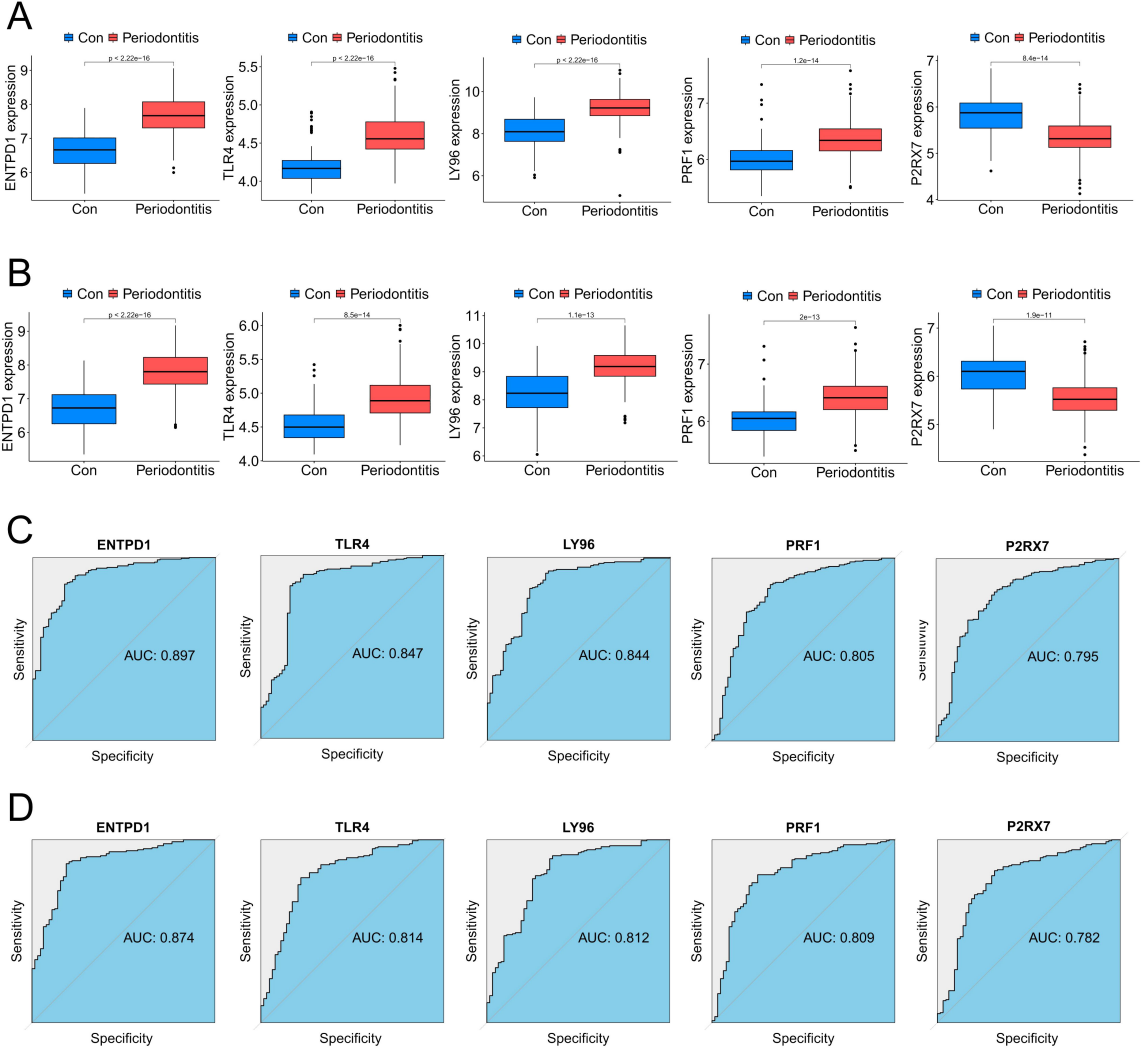
Expression pattern validation and diagnostic value. **(A)** Expression of ENTPD1, TLR4, LY96, PRF1 and P2RX7 in the the periodontitis database GSE16134 database. **(B)** Expression of ENTPD1, TLR4, LY96, PRF1 and P2RX7 in the the periodontitis database GSE10334 database. **(C)** ROC curves of ENTPD1, TLR4, LY96, PRF1 and P2RX7 in the periodontitis GSE16134 database. **(D)** ROC curves of ENTPD1, TLR4, LY96, PRF1 and P2RX7 in the periodontitis GSE10334 database.

In order to further screen potential ICD-related feature genes in periodontitis, we chose two machine learning algorithms. Using the above 11 genes as inputs for LASSO regression and RF algorithms, 6 genes and 5 genes were obtained, respectively (**Figures 4A–D**). Ultimately, five intersecting genes were obtained (ENTPD1, TLR4, LY96, PRF1 and P2RX7) (**Figure 4E**). These genes were then used in the construction of models and the assessment of diagnostic efficacy. Notably, the ranger approach performed the best in the external validation set (**Figures 4F, G**), and it also performed the best in the training set with an AUC of 0.937 (**Figure 4H**). To verify the performance of these vital genes, we evaluated them using two datasets: the training set GSE16134 and the validation set GSE10334. In line with the findings of the validation set, our findings demonstrated that ENTPD1, TLR4, LY96, PRF1 were elevated in periodontitis patients while P2RX7 expression was decreased (**Figures 5A, B**). ROC curves revealed that in the training set GSE16134, the AUC values for the four genes ENTPD1, TLR4, LY96, PRF1 and P2RX7 were 0.897, 0.847, 0.844, 0.805 and 0.795 respectively (**Figure 5C**). Furthermore, all five of the genes’ AUC values in the validation set GSE10334 were greater than 0.7, indicating that they may have potential applications as diagnostic tools (**Figure 5D**).

### 3.6 Interaction analysis and enrichment analysis of model genes

**Figure 6:**
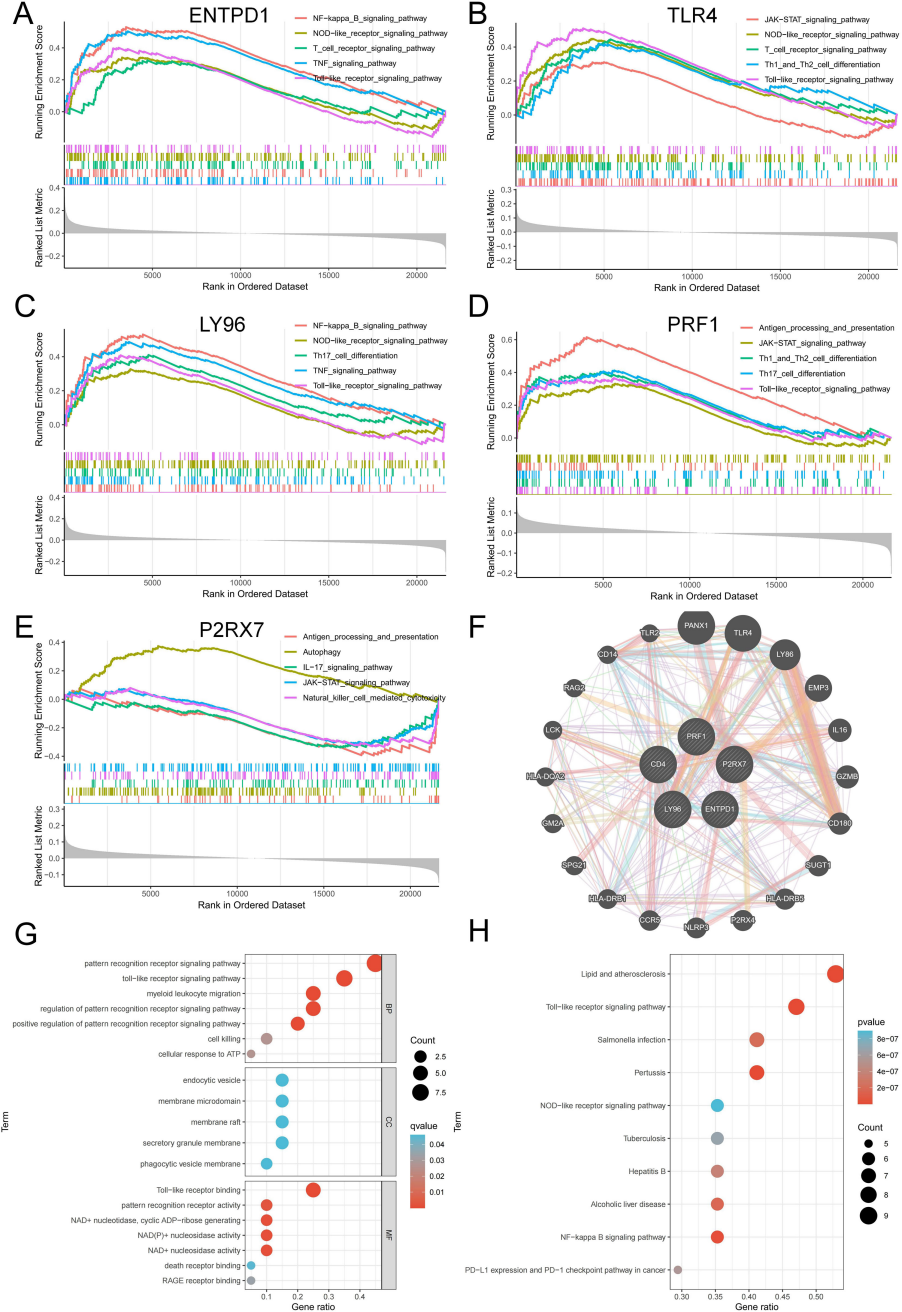
Interaction analysis of model genes and enrichment analyses. **(A-E)** GSEA identifies signaling pathways involved in ENTPD1, TLR4, LY96, PRF1 and P2RX7. **(F)** hub ICD-related genes co-expression network. **(G)** GO analysis of co-expressed genes. **(H)** KEGG analysis of co-expressed genes. ICD, Immunogenic cell death.

GSEA was utilized to examine the particular signal pathways connected to the hub ICD-related genes. ENTPD1, TLR4, LY96, and PRF1 were found to have positive associations with multiple ICD-related pathways, including antigen processing and presentation, toll-like receptor signaling pathway, NOD-like receptor signaling pathway, JAK-STAT signaling pathway, TNF signaling pathway, and T cell receptor signaling pathway (**Figures 6A–D**). P2RX7 showed a positive correlation with antigen processing and presentation, but a negative correlation with the IL-17 and JAK-STAT signaling pathways (**Figure 6E**). To find out more about the function of feature genes, we performed a GO/KEGG analysis of the top 20 genes based on connectedness using the GeneMANIA database (**Figure 6F**). The findings demonstrated that biological processes (BP) in this dataset were significantly enriched in the PRR signaling pathway, myeloid leukocyte migration, cell killing and cellular response to ATP, and MF were significantly enriched in PRR activity, toll-like receptor binding and death receptor binding (**Figure 6G**). KEGG showed that these genes were linked to a number of various ICD-related pathways, indicating that these genes may be involved in the occurrence of periodontitis through the regulation of ICD (**Figure 6H**).

### 3.7 Construction of the ICD subtype of periodontitis

**Figure 7:**
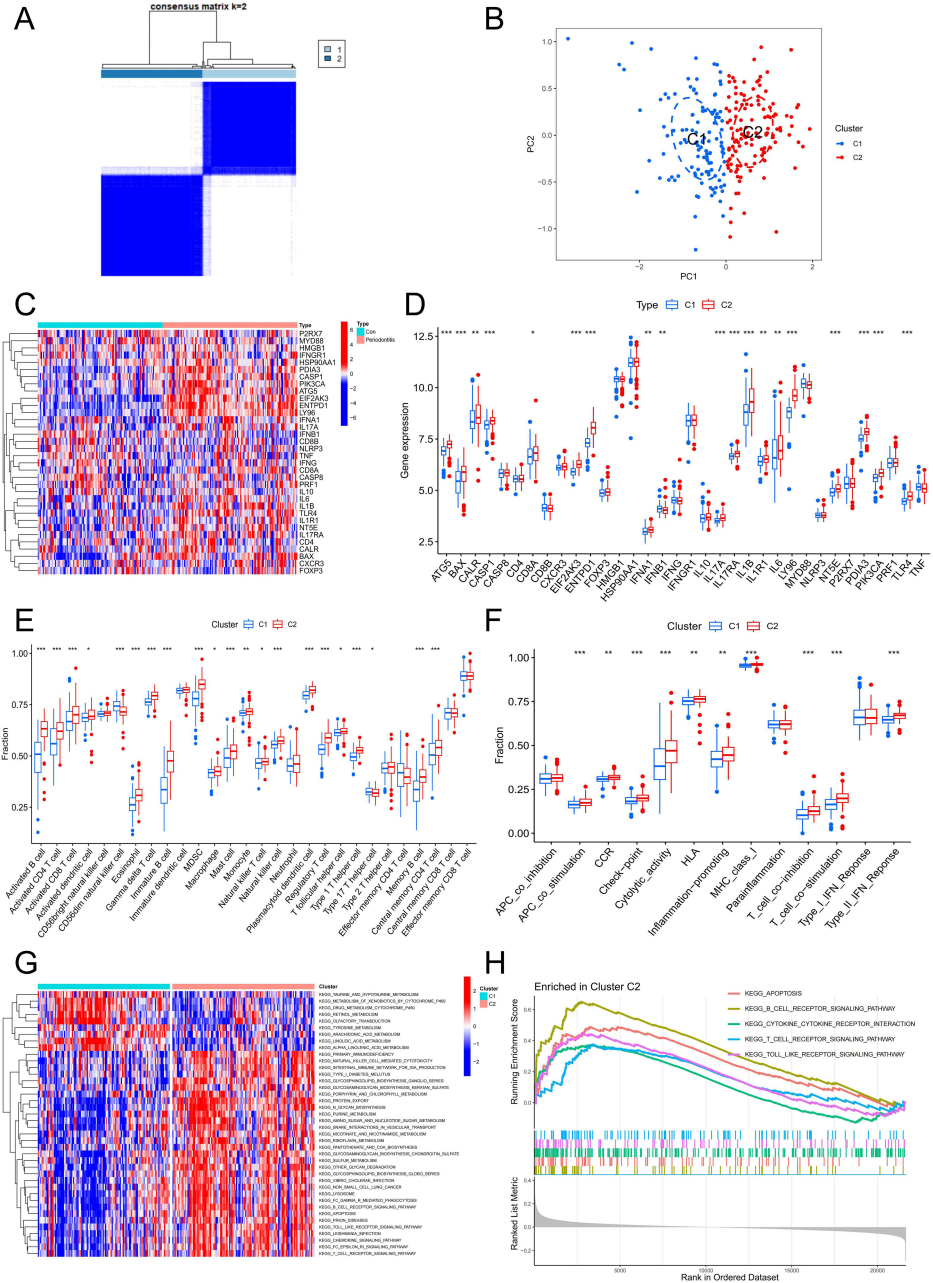
Identification of the ICD-related clusters in periodontitis. **(A)** Consensus matrix heatmap when k = 2. **(B)** PCA analysis of two ICD typing samples. **(C)** Heatmap of ICD-related genes in two clusters. **(D)** Boxplot of ICD-related genes in two clusters. **(E)** The fraction of immune cells infiltration in two clusters. **(F)** The fraction of immune function in two clusters. **(G)** GSVA analysis between two clusters. **(H)** GSEA analysis between two clusters. ICD, Immunogenic cell death.

A total of 241 periodontitis samples were clustered based on 5 hub genes. Consistency matrix analysis shows that k=2 is the ideal choice, and each sample in the cluster shows significant correlation cluster variables (**Figure 7A**). PCA analysis was carried out to determine the accuracy of the classification, and the findings demonstrated that it could reliably distinguish between clusters 1 and 2 (**Figure 7B**). **Figures 7C, D** revealed that the majority of genes associated with ICD were substantially up-regulated in C2, designating C2 as the group exhibiting high ICD expression and C1 as the group exhibiting low ICD expression. Subsequently, we conducted a thorough investigation on the features of the immune milieu associated with various ICD subtypes in periodontitis. A variety of immune cells were found to be up-regulated in the C2 group, especially T cells, B cells and a variety of APCs, which were intimately associated with ICD (**Figure 7E**). In addition, ICD-related immune functions such as APC co stimulation, MHC class I and T cell co stimulation were significantly up-regulated in the C2 group (**Figure 7F**). Additionally, GSVA and GSEA were used to investigate differences in the pathways linked to ICD subtypes. The findings demonstrated a considerable increase in apoptosis, the T cell receptor signaling pathway, the toll-like receptor signaling pathway, cytokine-cytokine receptor interaction, and natural killer cell-mediated cytotoxicity in the C2 group (**Figures 7G, H**). All of the findings pointed to the possibility that ICD can regulate the immunological milieu and, in turn, affect the development of periodontitis.

### 3.8 Experimental verification of ICD-related hub genes

**Figure 8:**
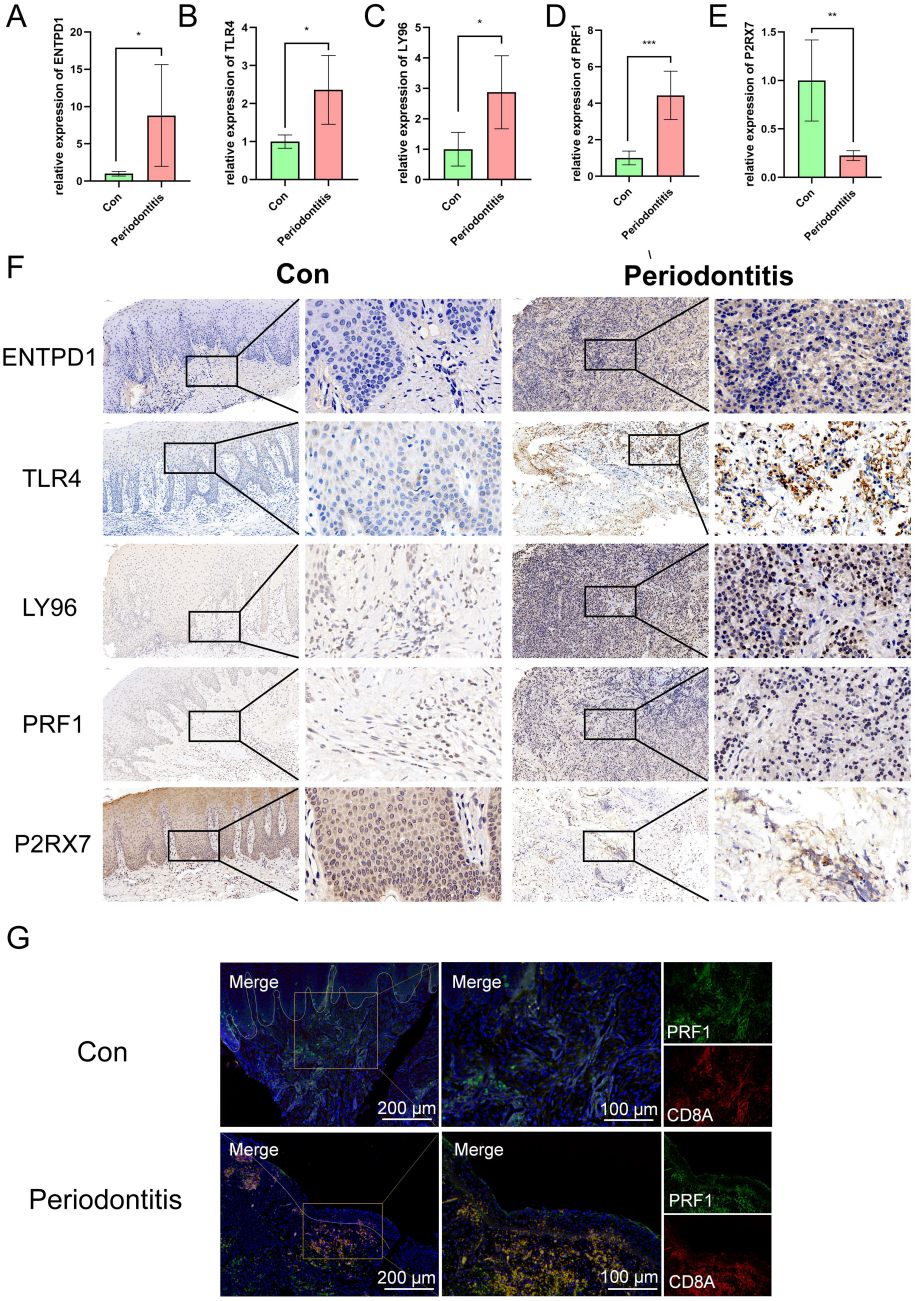
Expression of ENTPD1, TLR4, LY96, PRF1 and P2RX7 in patients with periodontitis and controls. **(A-E)** qRT-PCR results show the mRNA expression levels of ENTPD1, TLR4, LY96, PRF1 and P2RX7 in the gingivae of individuals in the healthy and periodontitis groups (n_con_=5, n_case_=5, respectively). GAPDH was used for normalization relative to the control group. **(F)** Immunohistochemistry staining of ENTPD1, TLR4, LY96, PRF1 and P2RX7 in the gingivae of individuals in the healthy and periodontitis groups. **(G)** Immunofluorescence staining of PRF1, in which CD8A positive represents CD8T cells. ICD, Immunogenic cell death; Con, control.

The veracity of the bioinformatics analysis results was further confirmed by immunohistochemistry and qPCR. In contrast to the healthy group, the periodontitis group had higher levels of ENTPD1, TLR4, LY96, and PRF1 expression and lower levels of P2RX7 expression, according to the qPCR data (**Figures 8A–E**). Immunohistochemical results were consistent with qPCR results, which confirmed the reliability of the ICD model (**Figure 8F**). Furthermore, we performed cell localization of PRF1 and found that it was mainly located in CD8T cells, and that both CD8T cells and PRF1 were abundantly activated in periodontitis samples, further confirming the existence of ICD (**Figure 8G**).

## 4 Discussion

An inflammatory chronic condition called periodontitis harms the tissues that support and surround teeth. People’s ability to chew may be affected if periodontitis is not treated in a timely manner, as it may result in tooth loss or loosening (37). In addition, periodontitis can also be a contributing factor in other systemic diseases like diabetes, RA, cancer and cardiovascular disorders (2, 3, 6, 38). As a result, it is necessary to research the pathophysiology of periodontitis. Meanwhile, the current research on the definition and diagnosis of periodontitis is not completely clear. Previous studies have used a range of clinical signs and symptoms to identify and diagnose periodontitis, such as exploratory bleeding (BOP), clinical attachment loss (CAL), and radiologically assessed alveolar bone loss (39). These clinical measurements have many limitations, despite the fact that they enable doctors to evaluate the severity and present extent of periodontitis as well as previous tissue loss. First, these clinical measures do not provide reliable data on biological activity or future disease course. In addition, when patients have these symptoms, the periodontal tissue has often been relatively serious damage. Finally, recording clinical measurements is subjective because it depends on the examiner. Given these limitations, Tonetti et al. (40) stress that biomarkers might be a useful tool for the early identification of periodontitis. GCF and saliva are the most widely used of these since they are simple and non-invasive to obtain, and their analysis is reasonably priced for the purpose of identifying biomarkers. In addition, bedside periodontitis diagnosis is feasible due to advances in biomarker detection technology (41). Consequently, biomarkers might prove crucial for the early detection of periodontitis in the future.

In this study, by combining the scRNA-seq and bulk-seq data, we found that a new cell death pattern called ICD may influence the onset of periodontitis, and fibroblasts may be the main source of ICD in periodontitis by scoring ICD-related gene sets in single cells, which was further verified by qPCR and IF staining. The role of fibroblasts in periodontitis has been confirmed. First, fibroblasts are an important part of periodontal tissue and are responsible for maintaining tissue structure and integrity. Secondly, fibroblasts can also initiate the inflammatory process that lead to bone loss and soft tissue destruction in periodontitis. Under inflammatory conditions, fibroblasts can produce a variety of chemokines such as CXCL1, CXCL2, CXCL12, CCL2, CCL19 and inflammatory mediators such as prostaglandin E2 (PGE2), matrix metalloproteinases (MMPs), and cytokines (IL-6 and IL-1β), which recruit and activate neutrophils and lymphocytes, thereby activating the immune response (42, 43). Furthermore, fibroblasts have also been found to be associated with DAMPs release. Studies have shown that LPS-stimulated fibroblasts initiate apoptosis and necrotic cell death to release HMGB1, which may contribute to the destruction of periodontal tissue (44, 45). In addition, ATP released by fibroblasts can also induce inflammatory cells to secrete cytokines and activate the expression of RANKL, triggering alveolar bone loss (28). These findings provide direction for our research, suggesting that fibroblasts may be able to activate immune responses by releasing DAMPs.

We analyzed the infiltration of immune cells and functions in periodontitis samples using the ssGSEA algorithm and explored the correlation between DEGs associated with ICD and immune cells and functions. The results unequivocally demonstrated the role of immunological factors in the pathophysiology of periodontitis, which may be connected to the development and control of ICD. In line with earlier research, patients with periodontitis showed a significant infiltration of immune cells, such as activated dendritic cells, CD56 bright natural killer cells, mast cells, and macrophages. This suggested the activation of a persistent pro-inflammatory response and the recruitment of inflammatory cells. We observed that the periodontitis group had a higher concentration of activated CD4 T cells, which led us to hypothesize that these cells might be engaged in immune killing during ICD development. Additionally, there was an increase in the quantity of ICD-associated activated CD8T cells, which can lead to severe tissue damage, resulting in severe and rapid loss of periodontal tissue (46). Similarly, multiple immune functions such as immune checkpoints, HLA, inflammation promotion, and T cell co-stimulation were activated in periodontitis samples, reflecting the importance of immune factors in periodontitis. Immunocorrelation results suggest that ICD-related genes may be involved in the pathogenesis of periodontitis by modulating a variety of immune cells and functions.

By analyzing bulk transcriptome data and using LASSO and RF algorithms, we obtained five ICD-related key genes (ENTPD1, TLR4, LY96, PRF1 and P2RX7). ROC curves, qPCR, and immunohistochemistry together highlight the potential of the five hub genes as biomarkers for the diagnosis of periodontitis. ENTPD1 (also known as CD39) is a member of the exonucleoside triphosphate diphosphate hydrolase family located on the surface of innate and adaptive immune cell subpopulations like monocytes, dendritic cells, and T/B cells (47). ENTPD1 is thought to play a significant role in immune system regulation, and the ENTPD1-Adenosinergic axis can act on human gingival fibroblasts to inhibit IL-1-induced matrix metalloproteinase-1 (MMP-1) expression (48). As a specific type I transmembrane receptor and PRR in the innate immune system, TLR4 plays a key role in acute inflammatory response, cell signal transduction and apoptosis (49). TLR4 on the DC can bind to released HMGB1 to accelerate the processing of phagocytic cargo in the DC and promote the presentation of antigens by the DC to T cells (50). In addition, TLR4 is a molecule necessary for LPS activation of target cells, and its activation can activate the innate immune system (51). Lymphocyte antigen 96 (LY96) is a key component required for Porphyromonas gingivalis to activate Toll-like receptor 4 (TLR4) with LPS (52). Periodontitis patients have significantly elevated levels of LY96, which leads to the formation of LY96-TLR4-CD14 complexes that trigger the myeloid differentiation factor-88 (MyD88) pathway, resulting in TNF-α, IL-6, IL-8, and IL-2 production in the gingival tissue (53). As one of the primary proteins of cytolysate granules, perforin (PRF1) is an important effector molecule in cytolysation mediated by T-cells and natural killer cells (54). It is essential to the development of immunological homeostasis, pathogen removal, and tumor surveillance as a certain indicator of immune cell killing capacity (55). P2X7R is a P2X receptor family member that is expressed on many immune cell types. Extracellular ATP, a critical regulator of inflammation, stimulates human periodontal ligament stem cells (PDLSCs) to produce more IL-1β and releases pro-inflammatory cytokines IL-8 and CCL20 via P2X7R (56). Furthermore, P2X7R expression can control osteoblast/osteoclast production and apoptosis at various phases to mediate bone metabolism (57, 58).

The study also has some limitations. First, our study relied primarily on publicly available scRNA-seq and bulk RNA-seq data, which lacked important variables such as clinical information on the severity, stage, or grade of periodontitis. This makes it extremely challenging for us to do a multi-angle analysis of the role of ICD in periodontitis, and the results may be biased. Therefore, future research is required to investigate the temporal dynamics of periodontal disease genesis and detection, specifically focusing on information about clinical disease characteristics and their relationship to ICD-based categories. Secondly, the precise regulatory mechanism of ICD in periodontitis and the relationship between key genes and phenotypes related to the disease are still lacking in-depth understanding. Further investigation of the specific role of ICD in periodontitis using *in vivo* and *in vitro* studies as well as the exploration of ICD-associated targeted therapeutic approaches will benefit the periodontitis research. Finally, we verified the results of bioinformatics analysis by qPCR and immunohistochemical staining. However, given the specificity of patient samples, the broad applicability of the biomarkers we identified still needs to be validated with large-scale clinical cohorts in the future.

## 5 Conclusion

In this study, we found that fibroblasts may be one of the major cells involved in ICD in periodontitis. Adaptive immunity initiated by ICD may be a contributing factor to the chronic inflammatory response in periodontitis. Five hub ICD-related genes (ENTPD1, TLR4, LY96, PRF1 and P2RX7) were identified for the construction of a diagnostic model for periodontitis. These ICD-related genes regulate the immune response, which may be a major factor in the pathophysiology of periodontitis. Summed up, these findings point to the possibility of creating novel periodontitis treatment plans by focusing on the molecular pathways and mechanisms linked to ICD.

## Data Availability

The original contributions presented in the study are included in the article/**Supplementary Material**. Further inquiries can be directed to the corresponding authors.
