# Supplementary material for: Analysis of immunogenic cell death in periodontitis based on scRNA-seq and bulk RNA-seq data
Source: Front Immunol. 2024 Nov 1;15:1438998. doi: 10.3389/fimmu.2024.1438998 (PMC11568468; doi:10.3389/fimmu.2024.1438998)
Supplement: Supplementary file 1 [file Image1.pdf]

Supplementary Material

A

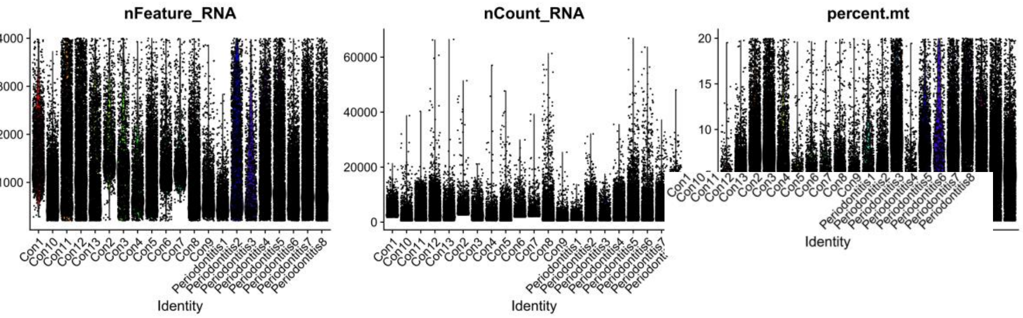

B

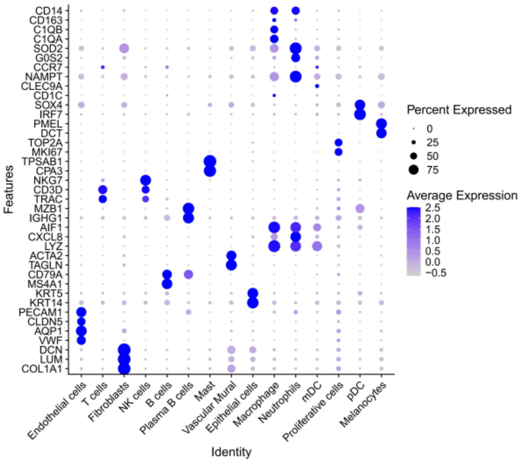

Supplementary Figure 1. Analysis of RNA-seq data. (A) Data quality control. (B) Gene markers used to annotate cell clusters.
