# Supplementary material for: Analysis of immunogenic cell death in periodontitis based on scRNA-seq and bulk RNA-seq data
Source: Front Immunol. 2024 Nov 1;15:1438998. doi: 10.3389/fimmu.2024.1438998 (PMC11568468; doi:10.3389/fimmu.2024.1438998)
Supplement: Supplementary file 2 [file Image2.pdf]

Supplementary Material

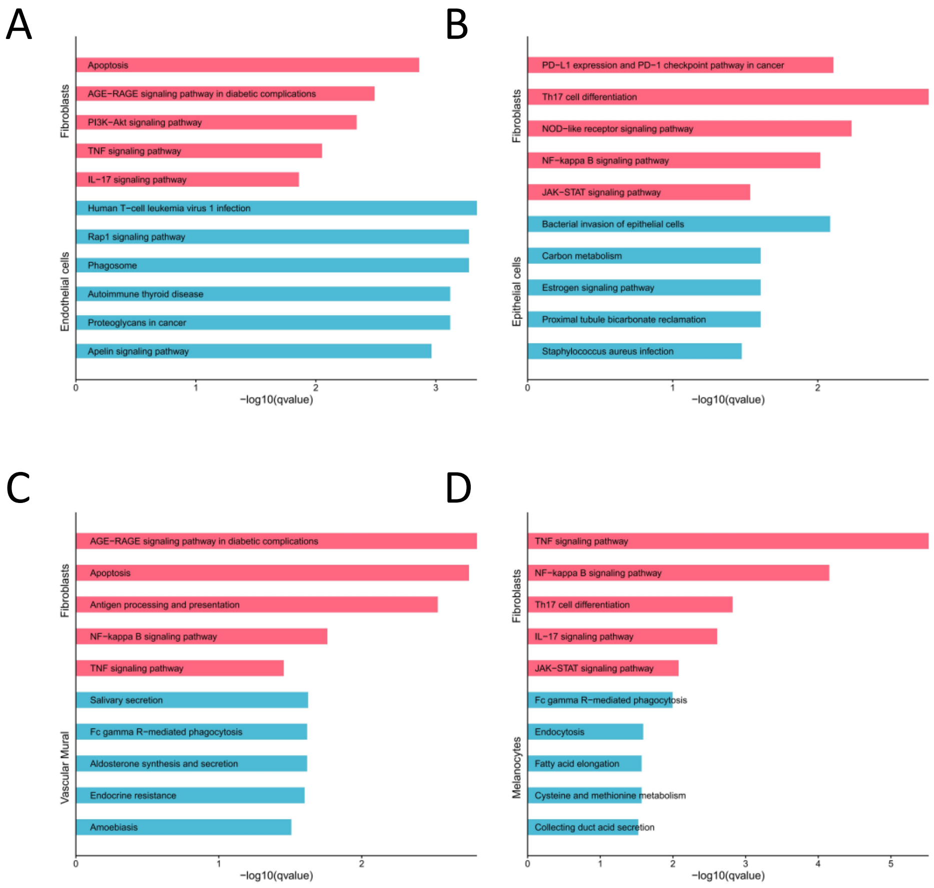

Supplementary Figure 2. Differential pathway analysis between fibroblasts and other non-immune cells (A) KEGG analysis of differentially expressed genes between fibroblasts and endothelial cells. (B) KEGG analysis of differentially expressed genes between fibroblasts and epithelial cells. (C) KEGG analysis of differentially expressed genes between fibroblasts and vascular murals. (D) KEGG analysis of differentially expressed genes between fibroblasts and melanocytes. logfc.threshold = 0.25, p_val_adj <0.05 were compared using KEGG analysis.
