# Supplementary material for: Analysis of immunogenic cell death in periodontitis based on scRNA-seq and bulk RNA-seq data
Source: Front Immunol. 2024 Nov 1;15:1438998. doi: 10.3389/fimmu.2024.1438998 (PMC11568468; doi:10.3389/fimmu.2024.1438998)
Supplement: Supplementary file 3 [file Table1.docx]

Supplementary Material

| Gene | Forward 5’-3’ | Reverse 5’-3’ |
| --- | --- | --- |
| GAPDH | CTTTGGTATCGTGGAAGGACTC | GTAGAGGCAGGGATGATGTTCT |
| HMGB1 | TATGGCAAAAGCGGACAAGG | CTTCGCAACATCACCAATGGA |
| ANXA1 | ACTGCTTCTACAGGATTTATGGTT | CAAAAAGCAGCCCCCATCAC |
| CXCL1 | AGTCATAGCCACACTCAAGAATGG | GATGCAGGATTGAGGCAAGC |
| CCL2 | AGAATCACCAGCAGCAAGTGTCC | TCCTGAACCCACTTCTGCTTGG |
| CXCL10 | ACCTCCAGTCTCAGCACCATGA | TGCAGGTACAGCGTACAGTTCT |
| ENTPD1 | CAGCCTTGGGAGGAGATAAA | GAGAGAGGTGTGGACAATGGTT |
| TLR4 | CCAGCCTCCTCAGAAACAGA | TCCCTCCAGCAGTGAAGAAG |
| LY96 | GAAGCAGTATTGGGTCTGCAA | TTGGAAGATTCATGGTGTTGACA |
| PRF1 | ACCTTCATCCAAGCATGGGG | TATTGTCCCACACGGTGCTC |
| P2RX7 | TCTTCCGAGAAACAGGCGAT | CCAACGGTCTAGGTTGCAGT |

**Supplementary Table 1.** Sequences of the primers used for RT-qPCR.
