# Supplementary material for: Analysis of immunogenic cell death in periodontitis based on scRNA-seq and bulk RNA-seq data
Source: Front Immunol. 2024 Nov 1;15:1438998. doi: 10.3389/fimmu.2024.1438998 (PMC11568468; doi:10.3389/fimmu.2024.1438998)
Supplement: Supplementary file 4 [file Table2.docx]

Supplementary Material

**Supplementary Table 2.** The number of each cell in the scRNA-seq analysis.

| **Sample** | **Endothelial cells** | **T cells** | **Fibroblasts** | **NK cells** | **B cells** | **Plasma B cells** | **Mast** | **Vascular Mural** | **Epithelial cells** | **Macrophage** | **Neutrophils** | **mDC** | **Proliferative cells** | **Melanocytes** |
| --- | --- | --- | --- | --- | --- | --- | --- | --- | --- | --- | --- | --- | --- | --- |
| **Con1** | 664 | 61 | 622 | 20 | 19 | 6 | 23 | 84 | 56 | 108 | 20 | 19 | 20 | 6 |
| **Con2** | 826 | 21 | 300 | 11 | 1 | 10 | 42 | 82 | 87 | 76 | 9 | 14 | 17 | 29 |
| **Con3** | 959 | 186 | 231 | 94 | 11 | 11 | 108 | 176 | 115 | 42 | 17 | 13 | 11 | 9 |
| **Con4** | 660 | 221 | 424 | 100 | 18 | 27 | 68 | 130 | 64 | 28 | 26 | 7 | 10 | 6 |
| **Con5** | 2313 | 360 | 3843 | 127 | 39 | 121 | 239 | 549 | 684 | 261 | 51 | 40 | 87 | 25 |
| **Con6** | 1076 | 42 | 813 | 10 | 1 | 52 | 26 | 76 | 303 | 74 | 18 | 10 | 37 | 14 |
| **Con7** | 613 | 26 | 373 | 17 | 0 | 9 | 0 | 133 | 90 | 51 | 3 | 5 | 7 | 10 |
| **Con8** | 2428 | 1600 | 1712 | 536 | 429 | 178 | 361 | 622 | 622 | 379 | 116 | 136 | 43 | 4 |
| **Con9** | 2015 | 560 | 833 | 190 | 3 | 89 | 35 | 345 | 154 | 97 | 12 | 22 | 16 | 3 |
| **Con10** | 2223 | 734 | 2012 | 310 | 47 | 139 | 370 | 487 | 751 | 261 | 78 | 52 | 54 | 38 |
| **Con11** | 1486 | 453 | 813 | 107 | 18 | 69 | 137 | 715 | 539 | 103 | 23 | 49 | 16 | 27 |
| **Con12** | 1392 | 1511 | 519 | 582 | 873 | 601 | 144 | 317 | 803 | 239 | 58 | 98 | 77 | 8 |
| **Con13** | 854 | 513 | 384 | 132 | 91 | 770 | 89 | 100 | 148 | 59 | 31 | 36 | 26 | 4 |
| **Periodontitis1** | 364 | 745 | 258 | 261 | 87 | 468 | 118 | 68 | 20 | 184 | 70 | 53 | 48 | 5 |
| **Periodontitis2** | 594 | 92 | 436 | 36 | 33 | 127 | 58 | 221 | 145 | 71 | 107 | 21 | 14 | 1 |
| **Periodontitis3** | 124 | 243 | 78 | 108 | 8 | 50 | 98 | 65 | 148 | 17 | 13 | 9 | 7 | 1 |
| **Periodontitis4** | 1199 | 520 | 1088 | 253 | 59 | 511 | 113 | 474 | 322 | 139 | 140 | 27 | 43 | 6 |
| **Periodontitis5** | 1127 | 985 | 662 | 335 | 131 | 1555 | 40 | 256 | 145 | 120 | 60 | 72 | 65 | 0 |
| **Periodontitis6** | 565 | 1101 | 476 | 443 | 395 | 1953 | 48 | 180 | 17 | 201 | 145 | 64 | 58 | 0 |
| **Periodontitis7** | 1755 | 2160 | 1430 | 745 | 204 | 929 | 142 | 254 | 51 | 205 | 90 | 76 | 60 | 19 |
| **Periodontitis8** | 715 | 1205 | 541 | 401 | 296 | 1829 | 188 | 444 | 62 | 263 | 305 | 123 | 87 | 2 |
| **Total(Con)** | 17509 | 6288 | 12879 | 2236 | 1550 | 2082 | 1642 | 3816 | 4416 | 1778 | 462 | 501 | 421 | 183 |
| **Total(Periodontitis)** | 6443 | 7051 | 4969 | 2582 | 1213 | 7422 | 805 | 1962 | 910 | 1200 | 930 | 445 | 382 | 34 |
| **Means(Con)** | 1346.846 | 483.692 | 990.692 | 172.000 | 119.231 | 160.154 | 126.308 | 293.538 | 339.692 | 136.769 | 35.538 | 38.538 | 32.385 | 14.077 |
| **Means(Periodontitis)** | 805.375 | 881.375 | 621.125 | 322.750 | 151.625 | 927.750 | 100.625 | 245.250 | 113.750 | 150.000 | 116.250 | 55.625 | 47.750 | 4.250 |
| **std deviations(Con)** | 680.218 | 527.734 | 1011.204 | 191.132 | 254.053 | 241.999 | 124.114 | 229.342 | 292.624 | 110.073 | 32.274 | 38.920 | 26.053 | 11.608 |
| **std deviations(Periodontitis)** | 525.069 | 652.294 | 441.187 | 219.105 | 136.643 | 760.970 | 50.808 | 151.551 | 100.896 | 79.733 | 87.632 | 36.824 | 26.499 | 6.364 |
| **P Value(Con VS Periodontitis)** | 0.070 | 0.141 | 0.344 | 0.113 | 0.745 | 0.003 | 0.587 | 0.605 | 0.050 | 0.772 | 0.007 | 0.332 | 0.208 | 0.041 |
